# Effect of smartphone apps on glycemic control in young patients with type 1 diabetes: A meta-analysis

**DOI:** 10.3389/fpubh.2023.1074946

**Published:** 2023-03-30

**Authors:** Linhua Pi, Xiajie Shi, Zhen Wang, Zhiguang Zhou

**Affiliations:** National Clinical Research Center for Metabolic Diseases, Key Laboratory of Diabetes Immunology (Central South University), Ministry of Education, Department of Metabolism and Endocrinology, The Second Xiangya Hospital of Central South University, Changsha, Hunan, China

**Keywords:** smartphone apps, type 1 diabetes, young patients, glycemic control, hypoglycemia

## Abstract

**Objectives:**

Achieving glycemic control is a great challenge for young patients with type 1 diabetes (T1D), especially during the transition from childhood to adulthood. As various smartphone apps have been developed to improve glycemic control in T1D, we performed a meta-analysis of randomized controlled trials to assess the effect of smartphone apps on glycemic control in young patients with T1D.

**Methods:**

We systematically searched PubMed, Embase, and the Cochrane Library for randomized controlled trials comparing combined usual care and smartphone app treatment to usual care alone. This meta-analysis is reported in accordance with the Preferred Reporting Items for Systematic Reviews and Meta-Analysis (PRISMA) statement. The primary outcomes were the weighted difference in means (WMD) of HbA1c change from baseline and the person-years incidence of mild hypoglycemia or severe hypoglycemia between intervention and control groups. We assessed pooled data by use of a random-effects model.

**Results:**

Of 1,190 identified studies, nine were eligible and included in our analysis (*N* = 748 participants). Relative to the control, using smartphone apps yielded a non-significant reduction in glycated hemoglobin (HbA1c) (WMD = −0.26, 95% CI: −0.56 to 0.05; *p =* 0.10) and no increased frequency of mild hypoglycemia (WMD = 1.87, 95% CI: −1.52 to 5.27; *p* = 0.49) or severe hypoglycemia (WMD = −0.04, 95% CI: −0.35 to 0.27; *p* = 0.80). In further subgroup analysis, compared with the recording-style app group, the auxiliary-style app group exhibited a significant reduction in HbA1c (WMD = −0.83, 95% CI: −1.10 to −0.56, *p* < 0.001).

**Conclusion:**

The current pooled data analysis did not reveal a significant reduction in HbA1c in young patients with T1D undergoing treatment with smartphone apps and usual care in combination. However, auxiliary-style apps with insulin or carbo calculators were beneficial in reducing HbA1c.

## Introduction

Type 1 diabetes (T1D) has brought a great burden to young people worldwide, involving 1,211,900 patients under 20 years old globally in 2021 (IDF diabetes atlas 2021) (1). Poor glycemic control is definitely associated with long-term complications. However, achieving glycemic control is a great challenge to patients with T1D, especially to young patients during the transition from childhood to adulthood. The interference factors include poor cognition of diabetes (2), poor adherence to diabetes management (3, 4), nutritional needs for growth and development (5), and the responsibility shift (6) in this special transition period. Despite advancements in technology aiding the self-management of T1D over the past few decades, a considerable proportion of patients did not achieve optimal glycemic control (7). There is an urgent need for new strategies to improve glycemic control in young patients with T1D that are easily available and cost-effective.

In the context of more than 2.7 billion individuals in the world using smartphones (8) and approximately 0.5 billion people already using mobile apps for diet (9), physical activity (10), and chronic disease management (11–14), smartphone apps—representing a newly emerging technology—have demonstrated enormous potential to provide an effective tool aiding self-management of T1D. A few randomized controlled trials have been conducted to evaluate the effectiveness of smartphone apps on glycemic control among youth with T1D in the past few decades. Among these studies that used smartphone app interventions to assist in glucose control, medication adherence, weight loss, and quality of life, the results were inconsistent overall, despite promising results in some small-scale studies.

A few systematic reviews and meta-analyses have been conducted to evaluate the effectiveness of smartphone apps in diabetes management among adults with type 1 or type 2 diabetes mellitus (15–17). To the best of our knowledge, however, there is a lack of meta-analyses that have been performed targeting young patients with T1D. Although our objective was to assess the potential role of smartphone app interventions in the management of T1D in young patients, we recognized that individual studies might not be able to provide sufficient data on their own to affect practice. We, therefore, performed a meta-analysis of randomized controlled trials to establish the effect of smartphone app interventions on the key outcomes of glycemic control and hypoglycemia in young patients with T1D.

## Methods

This meta-analysis is reported in accordance with the Preferred Reporting Items for Systematic Reviews and Meta-Analysis (PRISMA) statement (18). It has been registered in the International Prospective Register of Systematic Reviews (number CRD42021290537).

### Data sources and literature search

The literature search was conducted in the PubMed, Embase, and Cochrane Library databases for relevant studies without time period restriction, and only studies written in English were included. Medical Subject Headings (MeSH) terms were used as follows: “smartphone apps,” “type 1 diabetes mellitus,” and “randomized controlled trials.” Meanwhile, manual searching was performed to find further relevant studies. The final electronic database search was performed on 18 February 2023.

### Study selection and data extraction

We regarded studies as eligible for inclusion if they were randomized clinical trials performed among children, adolescents, or young adults (ages ranging from 0 to 24 years old) with T1D, studies including compared treatment involving smartphone apps and usual care to treatment with usual diabetes care only, had at least 4 weeks’ duration of intervention, and reported changes in HbA1c. Exclusion criteria were as follows: observational and retrospective studies, studies with less than 4 weeks duration of intervention, studies that did not assess smartphone apps, studies with participants aged over 24 years old, studies with participants diagnosed with other types of diabetes, and studies with incomplete data.

We extracted the following data from each selected study: research designs, population demographics, trial duration, sample size, study duration, a summary of interventions, main outcomes, and findings. Two independent investigators (LP and XS) performed the literature search, study selection, data extraction, and quality assessment. Disagreements were resolved by a third investigator (ZZ).

### Statistical analysis

The primary outcomes were the mean change in HbA1c from baseline to study end (described as weighted mean difference, WMD) and the person-year incidence of mild and severe hypoglycemia between the smartphone app-usual care combination treatment group (intervention group) and the usual care-only group (control group). WMD was calculated using the inverse variance random-effects model. The overall effect size with 95% confidence intervals (CIs) was used to evaluate smartphone apps for glycemic control, and a value of *p* of <0.05 was considered statistically significant.

We assessed the possibility of publication bias by constructing a funnel plot of each trial’s effect size against the standard error. The “one study removed” approach was performed to evaluate the influence of each study on the overall pooled estimate. We used the Cochrane *Q*-test to assess heterogeneity between studies. We also performed *I*^2^ testing to assess the magnitude of the heterogeneity between studies, with values greater than 50% regarded as being indicative of moderate-to-high heterogeneity. Subgroup analysis was conducted to explore the source of heterogeneity, including smartphone app style and the duration of trials. We used RevMan (version 5.3) for all statistical analyses.

## Results

### Study selection

**Figure 1:**
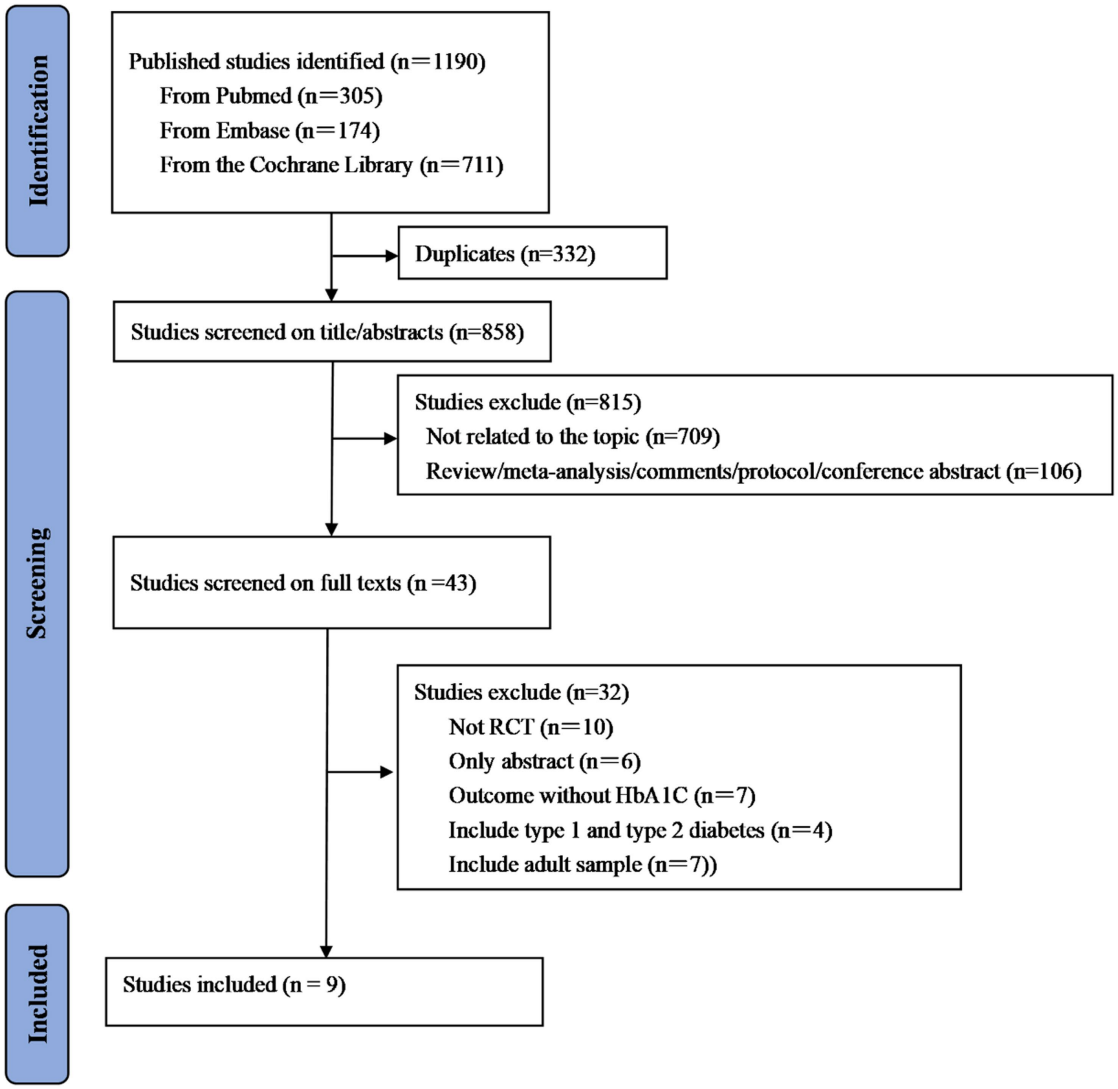
PRISMA flow diagram of the study search and selection.

A total of 1,190 studies were initially identified from PubMed, Embase, and the Cochrane Library. Then, 332 studies were removed because of duplications. After further screening based on title and abstract, 858 studies were excluded due to the lack of relevance to our topic or other reasons. A total of 43 studies were eligible for subsequent full-text review, and nine studies were ultimately included in the meta-analysis. The detailed process of the literature selection is illustrated in Figure 1.

### Study characteristics

All nine included studies were randomized controlled trials published between 2014 and 2021 (most in the last 5 years except for one in 2014) (Table 1). Sample sizes ranged from 32 to 168 among studies. Study durations varied from 4 weeks to 12 months (five trials with a duration ≥6 months). Participants were located in Canada, the United States, Germany, Denmark, Greece, Switzerland, Italy, and China. The mean age ranged from 12.65 to 17.8 years, and the maximum age was 24 years.

**Table 1:** Characteristics of the included studies.

First author	Year	Country	Sample size (recruited/completed)	Male%	Mean age (SD) (years)	Study duration	Duration of disease (SD) (years)	Intervention group	Control group
Alfonsi (19)	2020	Canada	I: 22/21	I: 50%	I: 13.98 (1.57)	3 months	I: 6.08 (4.14)	Carbohydrate count calculation using app	Routine carbohydrate count calculation
			C: 22/22	C: 73%	C: 13.98 (1.76)		C: 6.44 (4.45)		
Berndt (20)	2014	Germany	I: 34/34	I: 62%	I: 12.9 (2.0)	4 weeks	I: 5.0 (3.7)	App +Conventional therapy	Conventional therapy
			C: 34/34	C: 56%	C: 13.2 (2.9)		C: 5.3 (4.0)		
Castensøe-Seidenfaden (21)	2018	Denmark	I: 76/76	I: 42%	I: 17.6 (2.6)	12 months	I: 8.3 (4.3)	App +Usual outpatient care	Usual outpatient care
			C: 75/75	C: 51%	C: 17.6 (2.7)		C: 7.7 (4.7)		
Chatzakis (22)	2019	Greece	I: 40/40	I: 52.5%	I: 13.8 (3.0)	12 months	I: 6.7 (4.4)	Calculations using app	Routine calculations
			C: 40/40	C: 45%	C: 13.2 (2.7)		C: 6.1 (3.8)		
Goyal (23)	2017	Canada	I: 46/46;	I: 45.7%	I: 14.1 (1.7)	12 months	I: 7.1 (3.2)	App +Usual care	Usual care
			C: 46/45	C: 43.5%	C: 13.9 (1.5)		C: 6.6 (3.2)		
Hilliard (24)	2020	America	I: 55/54	41%	15.3 ± 1.5	12–16 weeks	NR^a^	App +Usual care	Usual care
			C: 25/24						
Klee (25)	2018	Switzerland	I: 16/16	NR^a^	NR^a^	3 months	NR^a^	App +Usual care	Usual care
			C: 16/16						
Bartolo (26)	2017	Italy	I: 92/86	I: 51.1%	I: 17.6 (3.1)	6 months	I: 8.6 (4.5)	Experimental glucose meter and telemedicine system	Traditional glucose meter
			C: 90/82	C:48.9%	C: 17.8 (3.0)		C: 9.0 (4.7)		
Xu (27)	2021	China	I:25/20	I:35%	I:12.65 (1.73)	6 months	I: 8.6 (4.5)	App +flash glucose monitoring	Flash glucose monitoring
			C:25/20	C:55%	C:13.35 (1.90)		C: 9.0 (4.72)		

^a^NR, not reported.

### Smartphone apps characteristics

The smartphone apps included in our analysis were complex in nature. They were mainly divided into two categories: auxiliary style and recording style. The former, which applied to three trials, was designed to assist in calculating carbohydrate content or insulin bolus; the latter, which applied to the other six trials, was designed to have one or more functions as follows: collecting biodata, tracking patterns or trends in diabetes management, self-monitoring, diabetes education, and social support in Table 2.

**Table 2:** Characteristics of mobile apps.

First author	Year	App style	Baseline mean HbA1c (SD)	Post-treatment Mean HbA1c (SD)	Real-time personalized feedback	HCP^g^ feedback
Alfonsi (19)	2020	Auxiliary^a^	I: 8.41 (1.84)	I: 8.06 (1.43)	No	No
			C: 8.35 (1.32)	C: 8.80 (1.60)		
Berndt (20)	2014	Recording^b^	I: 8.84 (1.71)	I: 8.12 (1.10)	Yes	Yes
			C: 8.96 (2.23)	C: 7.99 (1.26)		
Castensøe-Seidenfaden (21)	2018	Recording^c^	I: 9.6 (1.6)	I: 9.6 (1.7)	No	Yes
			C: 9.1 (1.4)	C: 8.8 (1.2)		
Chatzakis (22)	2019	Auxiliary^a,d^	I: 8.25 (0.8)	I: 7.2 (0.9)	No	No
			C: 7.9 (0.62)	C: 7.8 (0.7)		
Goyal (23)	2017	Recording^c^	I: 8.96 (0.7);	I: 8.96 (1.3)	No	No
			C: 8.92 (0.6)	C: 8.96 (1.2)		
Hilliard (24)	2020	Recording^c^	I: 9.1 (2.1);	I: 8.7 (1.7)	No	No
			C: 8.7 (2.1)	C: 8.4 (1.4)		
Klee (25)	2018	Auxiliary^d^	I: −0.33 (0.75)		Yes	Yes
			C: – 0.21 (0.79)			
Bartolo (26)	2017	Recording^c,e,f^	I: 9.9 (1.3)	I: 9.5 (1.4)	Yes	Yes
			C: 10.2 (1.5)	C: 9.8 (1.6)		
Xu (27)	2021	Recording^b,c,e,g,h^	I:7.78 (1.23)	I:7.2 (1.88)	Yes	Yes
			C:7.43 (2.21)	C:7.26 (0.89)		

^a^Calculating carbohydrate count; ^b^collecting biodata; ^c^tracking patterns or trends in diabetes management; ^d^calculating insulin bolus; ^e^blood glucose monitoring; ^f^social support; ^g^hospital care professional; ^h^diabetes education.

### Risk of bias assessment

**Figure 2:**
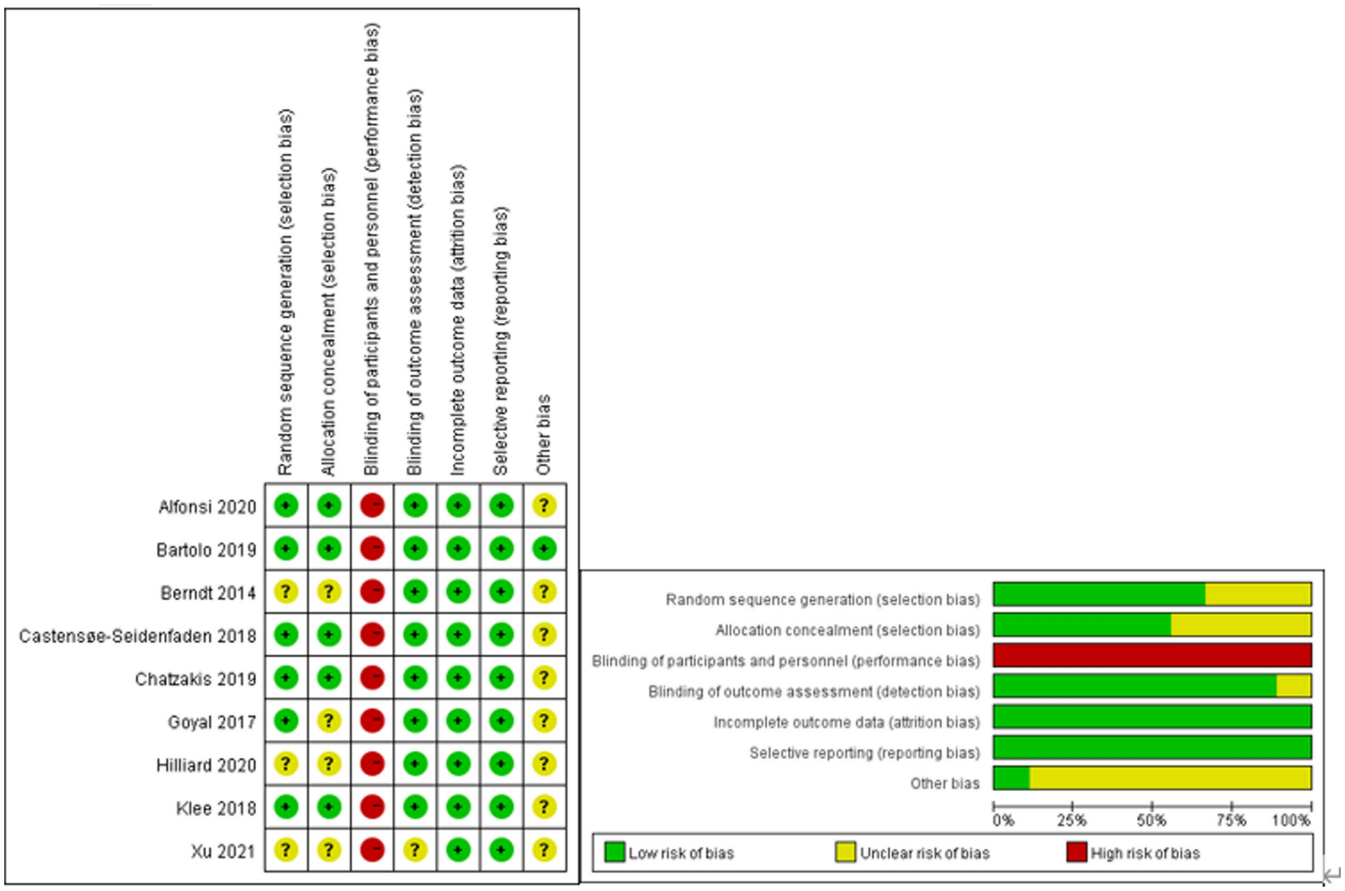
Risk of bias assessment.

The risk of bias was assessed by two independent reviewers with consultation from a third reviewer if necessary and is presented in Figure 2. All nine included studies showed overall fair levels of randomization. However, in three studies, there was no report of random sequence generation, and in four studies, allocation concealment was not reported.

### Smartphone app intervention and glycemic control

**Figure 3:**
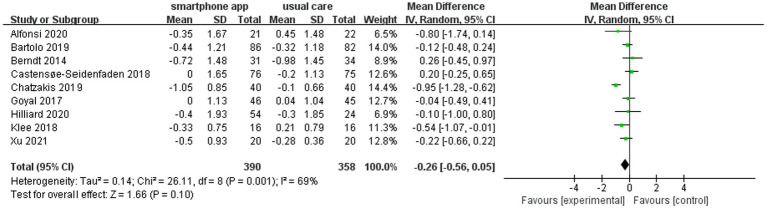
Forest plot of smartphone app intervention and change in HbA1c.

We defined the intervention and control groups as receiving smartphone apps and usual care combination treatment (*n* = 390) and only usual diabetes care (*n* = 358), respectively. The pooled analysis of nine included studies showed a non-significant reduction in HbA1c (WMD = −0.26, 95% CI: −0.56 to 0.05; *p =* 0.10) in the intervention group compared to the control group. Meanwhile, there was a moderate level of heterogeneity in the overall pooled effect (*I*^2^ = 69%) (Figure 3).

### Subgroup analysis

**Figure 4:**
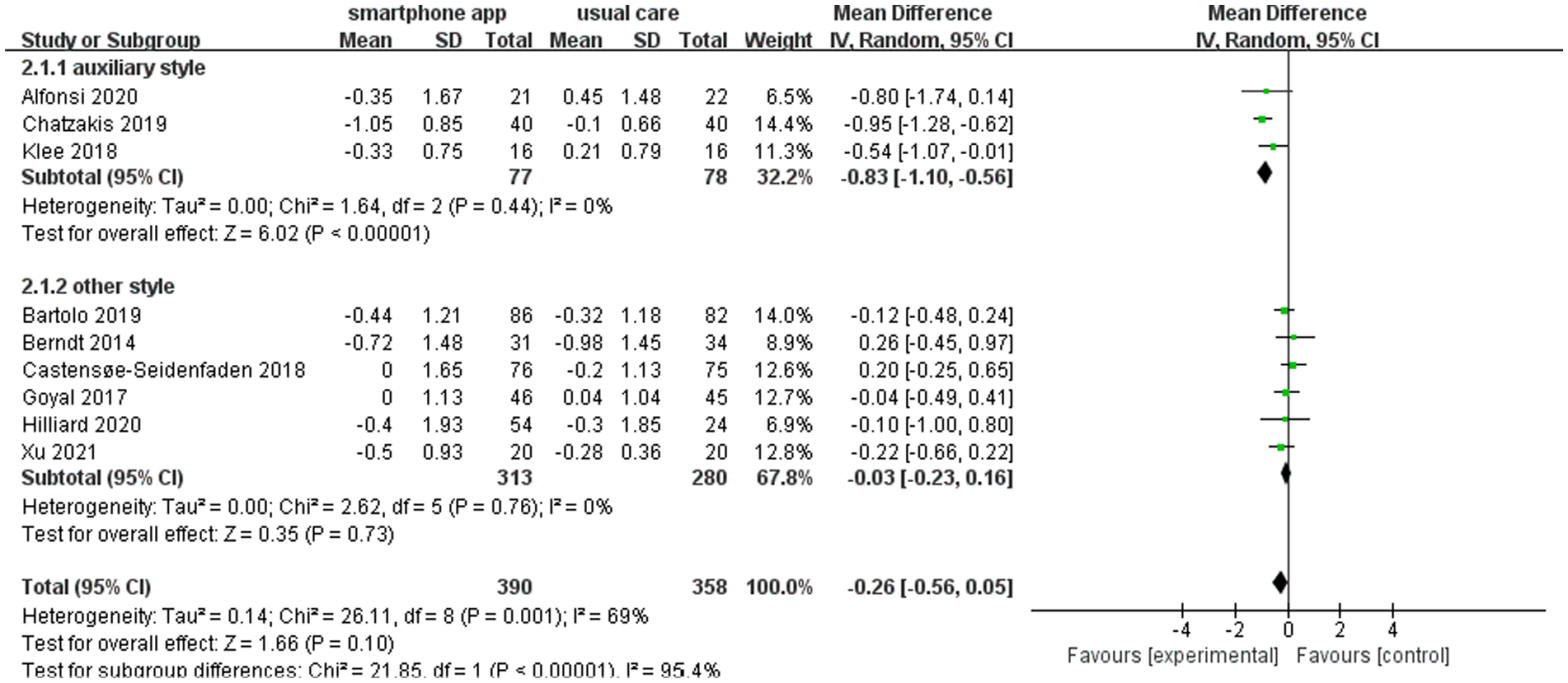
Forest plot of smartphone app interventions with different styles and changes in HbA1c.

Subgroup analysis was conducted to explore whether smartphone app style could affect glycemic control; auxiliary-style apps and recording-style apps were described more fully in previous studies. Pooled analysis of three studies showed that auxiliary-style apps yielded a significant reduction in HbA1c (WMD = −0.83, 95% CI: −1.10 to −0.56; *P* < 0.001, *I*^2^ = 0%) in the intervention group compared to the control group. The pooled analysis of six studies showed that recording-style apps exerted a similar effect on the reduction in HbA1c between the intervention group and the control group (WMD = −0.03, 95% CI: −0.23 to 0.16; *p* = 0.73, *I*^2^ = 0%) (Figure 4).

**Figure 5:**
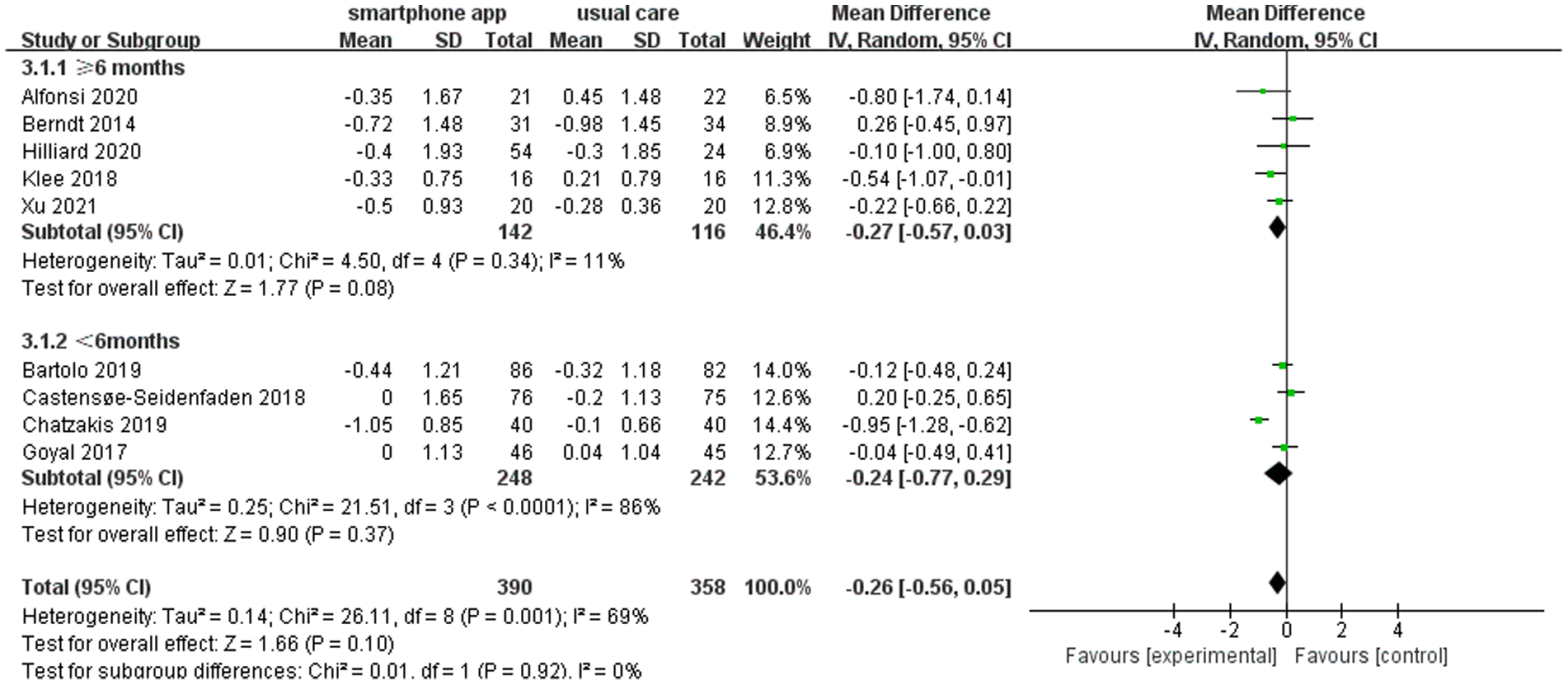
Forest plot of smartphone app interventions with different study durations and changes in HbA1c.

Another subgroup analysis was conducted to explore whether smartphone app intervention duration could affect glycemic control. The pooled analysis of five or more studies with intervention duration >6 months and four studies with intervention duration <6 months showed a non-significant reduction in HbA1c compared to their corresponding control groups (WMD = −0.27, 95% CI: −0.57 to 0.03, *p* = 0.08, *I*^2^ = 11%) and (WMD = −0.24, 95% CI: −0.77 to 0.29, *p* = 0.37, *I*^2^ = 86%), respectively (Figure 5).

### Sensitivity analysis

**Figure 6:**
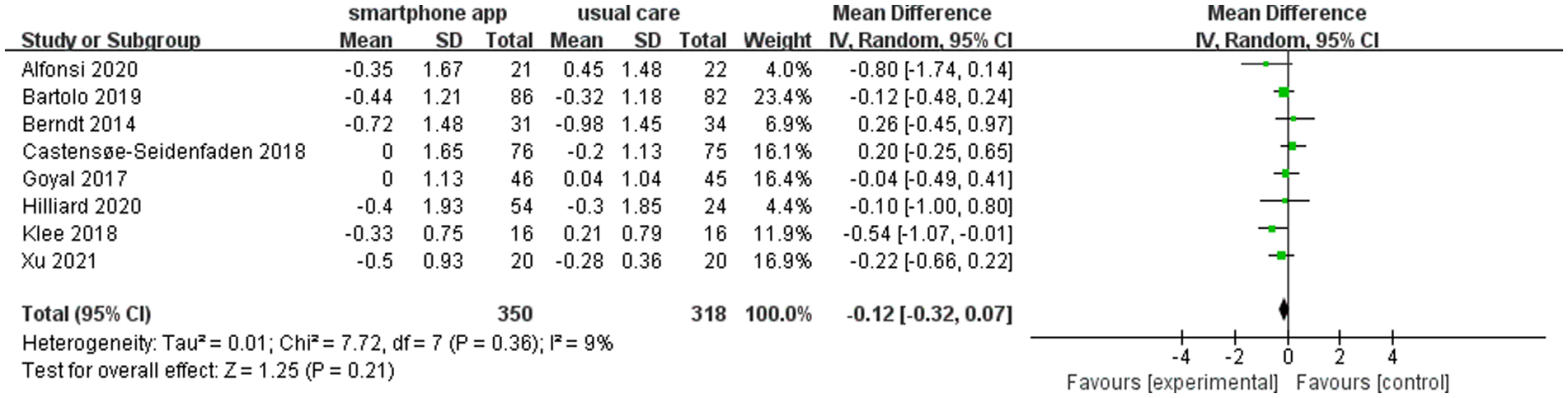
Forest plot of sensitivity analysis.

The heterogeneity assessment indicated a moderate level of heterogeneity in the overall pooled effect (*I*^2^ = 69%). The “one study removed” approach was used to assess the heterogeneity, and the removal of the Chatzakis et al. study (22) led to a reduced HbA1c change from baseline to study end between the intervention group and control group. However, it did not alter the final result. Meanwhile, the value of *I*^2^ became 9% (Figure 6).

### Risk of hypoglycemia

**Figure 7:**
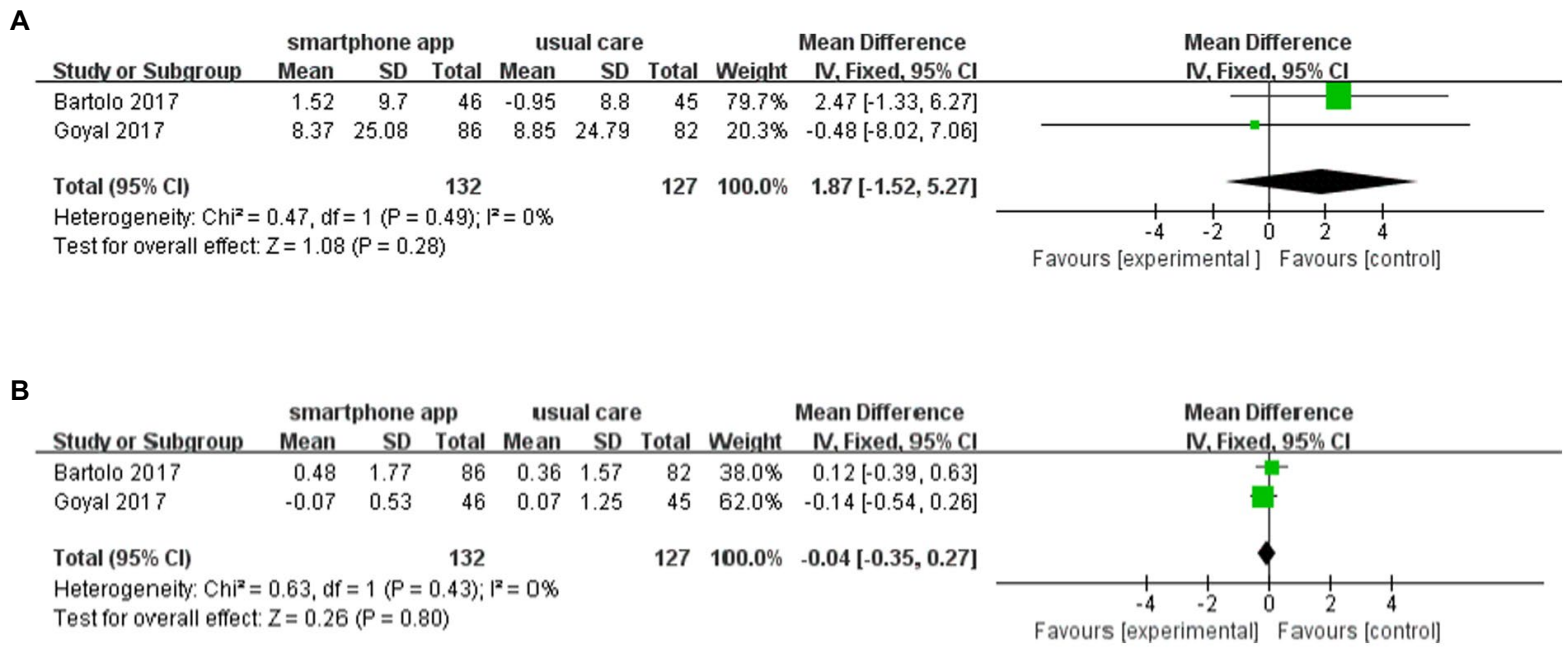
Forest plot of smartphone app intervention and incidence of **(A)** mild and **(B)** severe hypoglycemia.

Notably, six (21–23, 25–27) of nine included studies reported the risk of hypoglycemia during the intervention period. All six studies demonstrated that using smartphone apps did not increase the risk of hypoglycemia. Pooled data from two studies (*n* = 132 participants) showed no increased frequency of mild hypoglycemia of 1.87 (95% CI: −1.52 to 5.27, *p* = 0.28, *I*^2^ = 0%) (Figure 7A) or severe hypoglycemia of −0.04 (95% CI: −0.35 to 0.27, *p* = 0.80, *I*^2^ = 0%) (Figure 7B) in the intervention group compared to the control group.

### Publication bias

**Figure 8:**
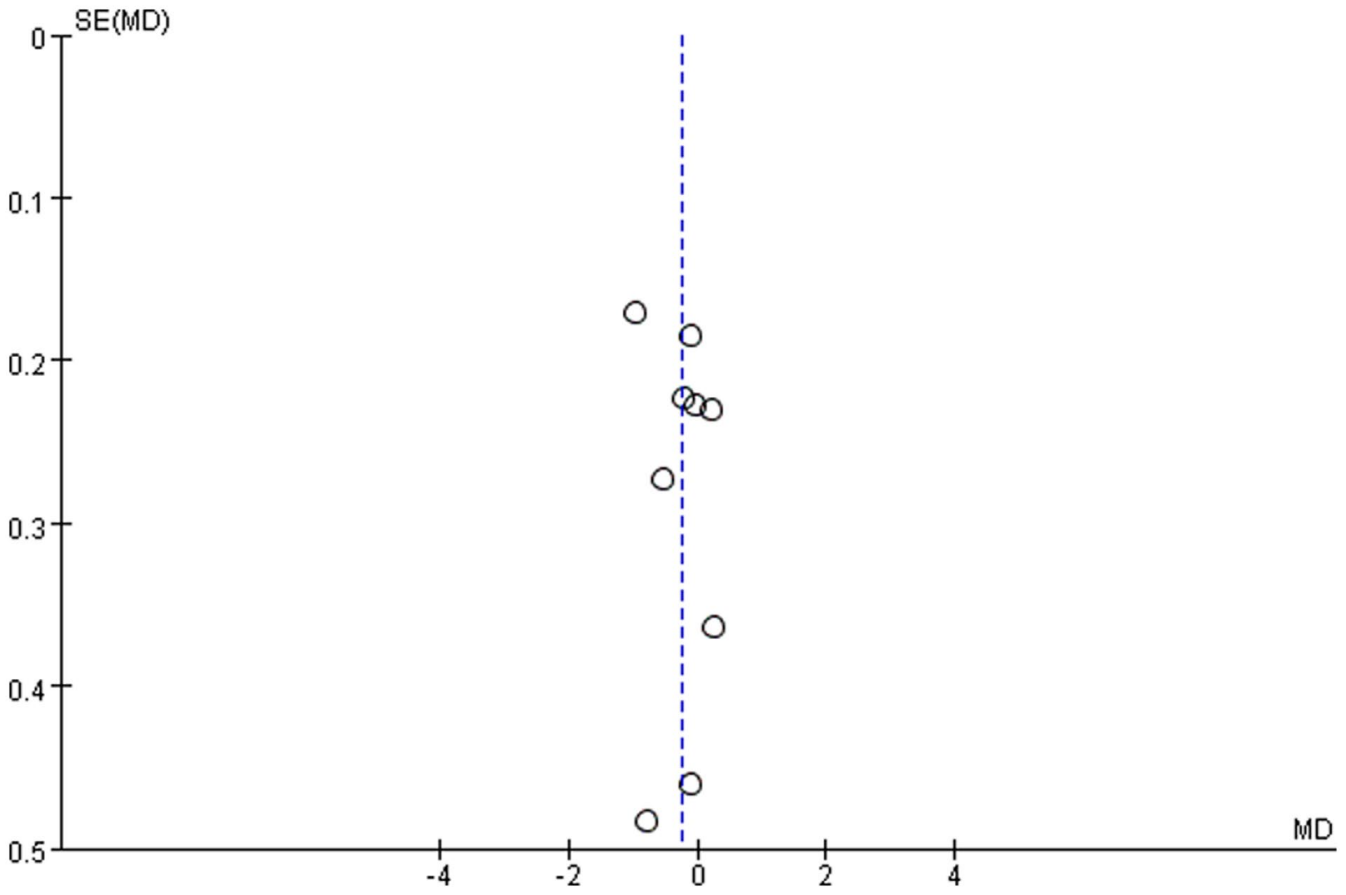
Funnel plot of publication bias assessment.

We assessed the possibility of publication bias by constructing a funnel plot from the analyses of the effect on glycemic control. As shown in Figure 8, more than half of the included studies were distributed around the top of the funnel plot, and it was relatively symmetric overall. Therefore, there was evidently no publication bias.

## Discussion

### Principal findings

A total of nine randomized controlled studies of fair quality were included in our study. The results showed that, compared with the control group, using smartphone apps can yield a trend in HbA1c from baseline to study reduction without an increased frequency of hypoglycemia, although not statistically significant. Furthermore, in the subgroup analysis to assess the effect of app style, compared with recording-style apps, auxiliary-style apps yielded a significant reduction in HbA1c. Meanwhile, in the subgroup analysis to assess the effect of intervention duration, it was found that compared to an intervention duration of less than 6 months, an intervention duration of more than 6 months was associated with a similar reduction in HbA1c.

### Public health implications

Achieving glycemic control plays a vital role in avoiding late longstanding complications, which is also a great challenge in T1D management. Patients with T1D are prone to require lifelong comprehensive management, which includes insulin therapy, metabolic monitoring, nutritional guidance, diabetes education, lifestyle management, and psychosocial care (28). Good adherence to the diabetes regimen would help to meet the target of metabolic control (29). Only 14% of youths with T1D, however, had met the glycemic control recommendation from the American Diabetes Association (29, 30), which might be attributed to the special period of maturation—the transition from childhood to adulthood—that involves a more important role of self-management and reduced engagement with caregivers (31, 32). In the context of more than 2.7 billion individuals in the world using smartphones (8) and approximately 0.5 billion people already using mobile apps for diet (9), physical activity (10), and chronic disease management (11–14), smartphone apps, a newly emerging technology, have demonstrated enormous potential to provide an effective tool aiding self-management of T1D. Different types of smartphone apps have been developed to assist self-management of T1D among youth, such as “iSpy” (19) and “Euglyca” (22). These are intended to improve glycemic control by encouraging glucose monitoring, assisting data collection, coaching people with diabetes, guiding healthy nutrition and medication dosing, and maintaining lifestyle modifications (33). Some small-scale trials of smartphone apps targeting glucose medication adherence, weight loss, and quality of life have shown promising results (34, 35).

### Comparison with other studies

A previous study indicated a consistent reduction in HbA1c in patients with type 2 diabetes mellitus, while in patients with T1D, the result was inconclusive (15). Patients with T1D receive advertisements regarding many kinds of apps to improve their treatment. It is important to know whether they are also useful in improving glycemic balance. The results of the present study suggest that apps with functional modularity containing carbohydrate counting or insulin dosage adjustment provide a generally positive improvement in HbA1c among young people with T1D. Nearly all individuals with T1D receive insulin, and insulin therapy is essential for optimal glucose control in patients with T1D (36, 37). The other pillar of T1D management is maintaining a healthy diet. Accurate counting of carbohydrates is paramount to the appropriate dosage of insulin, and regulating food intake and counting carbohydrates have a positive effect on metabolic control in children and adolescents with T1D (38). It is plausible that diabetes apps with functional modularity containing carbohydrate counting or insulin dosage are more effective. These characteristics deserve more consideration in future smartphone app design. While some smartphone apps have recording functions, the results regarding such functionality were inconsistent; further studies are needed to explore the effectiveness of this kind of smartphone app.

In addition, our results demonstrated no association between follow-up duration and reductions in HbA1c. This finding resonates with previous literature (39, 40). Lifelong self-management is needed for all age groups of patients with T1D; however, the longest follow-up duration in our study was 12 months, and the long-term effects of smartphone apps are still largely unknown.

Safety is one of the largest issues that limit the implementation of diabetes apps. Two studies compared the incidence of hypoglycemia between the smartphone app group and the control group, and the other studies reported the safety of the apps with descriptive words. Overall, smartphone apps were safe and associated with no increase in the number of hypoglycemia episodes.

### Strengths and limitations

The strengths of the analysis include a sensitive search to identify eligible trials and independent study identification, selection, data extraction, and adjudication of risk of bias by two reviewers. The limitations are as follows: (1) the sample size of individual studies, ranging from 16 to 86 in the intervention group, was relatively small, although all included trials were randomized controlled trials; (2) the optimal intervention duration was unknown, as lifelong self-management is needed for all age groups of patients with T1D; (3) as the included studies were mainly conducted in Europe (6/9), North America (2/9), and Asia (1/9), geographical or ethnic disparities should be considered for underlying bias; and (4) the overall heterogeneity was high, with an *I*^2^ of 69%. When we removed the study of Chatzakis et al. (22), the value of *I*^2^ decreased to 9%, which indicated that the high heterogeneity is mainly caused by this article with a great contribution. Meanwhile, a reduced HbA1c change from baseline to study end was found between the intervention and control groups, but it did not alter the final result.

## Conclusion

The current pooled data analysis did not reveal a significant reduction in HbA1c in young patients with T1D using smartphone apps and usual care in combination. Auxiliary-style apps with insulin or carbo calculators, however, provided evident benefit from HbA1c improvement in young patients with T1D. As smartphones have been increasingly widely used in daily life, auxiliary-style apps could provide a potential strategy for improving glycemic control in young patients with T1D. Further well-designed trials are required to verify efficacy and safety.

## Data availability statement

The original contributions presented in the study are included in the article/supplementary material, further inquiries can be directed to the corresponding authors.

## Author contributions

LP and XS independently conducted the literature search and screened the literature. ZZ resolved disagreements. LP analyzed data and wrote the manuscript. ZW and ZZ designed the study and revised the manuscript. All authors have read and approved the final version.

## Funding

This research was supported by the National Natural Science Foundation of China (No. 82000798), the Science and Technology Innovation Program of Hunan Province (No. 2022RC1010), and the Hunan Provincial Natural Science Foundation of China (No. 2022JJ30851).

## Conflict of interest

The authors declare that the research was conducted in the absence of any commercial or financial relationships that could be construed as a potential conflict of interest.

## Publisher’s note

All claims expressed in this article are solely those of the authors and do not necessarily represent those of their affiliated organizations, or those of the publisher, the editors and the reviewers. Any product that may be evaluated in this article, or claim that may be made by its manufacturer, is not guaranteed or endorsed by the publisher.
